# International nurse education leaders’ experiences of responding to the COVID‐19 pandemic: A qualitative study

**DOI:** 10.1111/jan.14892

**Published:** 2021-06-09

**Authors:** Robin Ion, Alison Craswell, Lynda Hughes, Amy Johnston, Lynn Kilbride, Natasha Hubbard‐Murdoch, Debra Massey

**Affiliations:** ^1^ University of the West of Scotland Paisley UK; ^2^ University of the Sunshine Coast Sippy Downs Queensland Australia; ^3^ Griffith University Gold Coast Queensland Australia; ^4^ Queensland University of Technology Brisbane Queensland Australia; ^5^ University of Dundee Dundee UK; ^6^ Saskatchewan Polytechnic Saskatoon Saskatchewan Canada; ^7^ Southern Cross University East Lismore New South Wales Australia

**Keywords:** COVID‐19, education, leadership, learning, pandemic

## Abstract

**Aims:**

To explore the experiences of strategic leads for nurse education as they sought to respond to the COVID‐19 pandemic.

**Design:**

We utilised a qualitative interpretative approach to explore education leaders’ experiences of leading during the early months of the pandemic.

**Methods:**

Nineteen leaders with significant strategic responsibility for nurse education in Australia, Canada, New Zealand, Singapore and the United Kingdom were identified via purposive sampling and agreed to participate. Interviews were held between May and July 2020.

**Results:**

Four overarching themes arose from the analysis: (1) Crisis driven adaptability & flexibility; (2) Responsive, complex and changing communication; (3) Making decisions for student and staff safety; (4) Looking to the future; stronger partnerships.

**Conclusion:**

Internationally, while nursing education leaders faced different problems, they shared a common goal amidst the crisis to remain student‐centred. They demonstrated they were able to face major challenges, respond to large scale logistical problems and make decisions under significant and ongoing pressure.

**Impact:**

In responding to the pandemic, nurse leaders shared knowledge and offered mutual support. This bodes well for future collaboration. The move to online learning accelerated an existing trend and it seems likely that this will continue. Given the pressures they experienced over an extended period, the sector may wish to consider how it prepares and supports existing and future leaders.

## 1 INTRODUCTION

Drawing on interviews with strategic leads for nurse education from Canada, Australia, New Zealand, Singapore and the UK, this paper explores the experiences of these leaders in the early months of the current COVID‐19 global pandemic. It provides insight into the decisions they made, the reasons given for these, their personal successes and regrets. Lessons that might be learned and taken forward as the COVID‐19 pandemic unfolds, and in the event of future pandemics are also considered.

At the time of writing, the WHO (2021) reports over one hundred and eight million cases of COVID‐19 worldwide, with over two million two hundred and ninety thousand deaths; with numbers continuing to rise. The International Council of Nurses’ (ICN) (2020) latest analysis shows that the number of nurses who have died after contracting COVID‐19 is over 2200. The economic impact of the virus is estimated to run into the equivalent of trillions of US dollars, jobs are being lost on a huge scale, local and national lockdowns are in situ and second and third waves of infection are now a reality in parts of Europe. In a world in which hyperbole is increasingly common, and where the meaning of words is not infrequently debased by their inappropriate use, this truly is an unprecedented event.

## 2 BACKGROUND

As the largest professional group in the global healthcare workforce (World Health Organization, 2020), nurses have been at the forefront of the healthcare response to the pandemic. To date, nurse academics have largely focussed their COVID‐19 related work on aspects of the patient, nurse or student experience (Gómez‐Ibáñez et al., 2020; Kuliukas et al., 2021; Nie et al., 2020; Watson & Hayter, 2020).

An exploration and associated critique of the role of nurses in the most senior leadership positions has also begun to emerge, for example, Rosser et al. (2020) questioned the ability of some nurse leaders to visibly influence national policy and public discussion of the crisis. These are echoed in Hayter and Jackson’s (2020) editorial which questions both the wisdom of deploying nursing students to fill perceived gaps in the workforce, as well as the planning of a coherent and viable response to the huge disruption caused to the student journey as a result of the pandemic. To date, the voices of those senior leaders charged with making decisions about nurse education during the crisis have, however, been silent.

There is considerable variation in the ways in which nurse education is organised across the world. Differences exist in relation to the clinical setting and the amount of time students are required to spend in clinical practice, as well as in the organisation and level of preparation programmes. The relationships between key stakeholders such as education providers, clinical partners, industry, regulatory bodies and government are also variable. In Canada and Australia, for example, the primary relationship with government is at state level, while in the UK it lies with central government. Despite these differences, there is a great deal of commonality. While the names of posts might vary from Dean to Head to Chief Nurse, those with strategic responsibility for nurse education are charged, albeit not exclusively, with ensuring educational and professional standards for nursing students, providing quality learning opportunities, delivering a steady stream of new registrants, while also making sure that all of this is undertaken in a manner which is supportive, engaging and cognisant of student health and well‐being. COVID‐19 has profoundly tested educational leads across the world in all these areas, particularly in relation to the provision and continuity of placement where “business as usual” was largely impossible. In the UK, programmes have been hugely disrupted with first year placements suspended from March 2020 until the beginning of 2021, while other students were offered the opportunity to temporarily give up supernumerary status in order to join the workforce—clinical placement for those who declined was suspended. In Canada, Australia and New Zealand, the response to risk was more nuanced with some students withdrawn from clinical placement, while others remained. With very few exceptions, face‐to‐face delivery of education ceased for most providers sometime in March 2020. A summary of the many difficulties faced by faculty in Canada because of such changes and adaptations has been provided by Dewart et al. (2020). They report colleagues struggling with balancing the risk to students and the community of allowing practicum to continue, the challenge of reassuring students about progression and the moral dissonance and distress sometimes associated with the subsequent decisions. Likewise, while Carolan et al. (2020) have explored some of the opportunities which might accrue as a consequence of the transformations initiated in nurse education in response to the pandemic, they are equally clear about the scale of the impact it has had on the sector.

In this paper, we explore experience of senior leaders involved in nursing education who were charged with directing and overseeing such changes, managing staff, making strategic decisions and responding to the unfolding pandemic. We sought to understand the difficulties they faced, how they negotiated these, and what lessons they learned. While there is a very strong case for also examining the role played by those with operational level leadership, for example, programme and course leads, our interest here was in the experience of those with strategic level responsibility, the decision makers. Typically, members of this group were directly responsible for faculty staff, student education and well‐being, and were accountable to state or national government and / or the national regulator. In many cases they reported directly to the most senior university managers, or to elected officials in government.

## 3 THE STUDY

### 3.1 Aims

To explore the experiences of strategic leads for nurse education as they sought to respond to the COVID‐19 pandemic.

### 3.2 Design

We utilised a qualitative interpretative approach to explore education leaders’ experiences of leading during the COVID‐19 pandemic. All authors are nurses and therefore ‘insiders’ or part of the social group under examination (Bonner & Tolhurst, 2002). Additionally, two authors inhabited dual roles as both participants and researchers (Probst, 2016).

### 3.3 Participants

Thirty‐two leaders with significant strategic responsibility for nurse education in Australia, Canada, New Zealand, Singapore and the United Kingdom were identified via purposive sampling and approached via email to participate in the study. Thirteen nurse education leaders who were approached did not respond to the email inviting them to participate. The participant group included those whose primary role was university based, along with others who worked in government and professional regulation around nursing education. Two authors were also participants in the research. Their interviews were undertaken by a member of the team they had not met and who was not part of the initial research conceptualisation.

### 3.4 Data collection

Data were collected remotely between May and July 2020 via individual semi‐structured, digitally recorded interview. This facilitated compliance with social distancing requirements and allowed for data collection from geographically distant informants. All authors (six females, one male) undertook interviews. Each interview lasted approximately 45 min. Study information and a copy of the interview schedule were provided in advance of interviews and consent for digital recording was sought. Participants were asked to relate their experiences of and reflections upon their role as strategic leads and decision makers for nurse education during the pandemic. Interviews were professionally transcribed, and transcriptions checked against the voice recording for accuracy by the research team. Written consent was obtained prior to interviews.

### 3.5 Ethical considerations

Ethical approval was obtained from the University of the West of Scotland (12005). This article was prepared in line with the COREQ checklist for reporting qualitative studies (Tong et al., 2007).

### 3.6 Data analysis

Analysis was conducted in line with Braun and Clarke’s (2006) six step process. In stage one—familiarisation with the data corpus, audio‐recordings were listened to by four members of the team, while field notes taken at the time of the interviews were also reviewed. At this point further notes were taken, and initial codes were assigned to the texts by all four members. Introductory ideas and thoughts were then shared and refined across the wider research team. In stage two, a detailed review of the data and preliminary themes were undertaken. Further codes were identified and developed. In stage three, codes were re‐examined by two team members and, where appropriate, used to develop candidate themes. In stage four these candidate themes were again reviewed for integrity, coherence and depth by three of the authors. In the next stage, five themes were further developed and shared with all authors for discussion, refinement and confirmation. In stage six, themes were further developed, merged and finalised.

### 3.7 Validity and reliability/rigour

We drew on the work of Braun and Clarke (2006) in order to ensure trustworthiness and rigour in our data collection and analysis. Data were digitally recorded and professionally transcribed to ensure accuracy and to enable review by the full team, all of whom have experience in qualitative work. Coding and theme development was carried out and checked by at least two team members as indicated above. Disagreements were resolved through discussion data examination. Regular team meetings were held to discuss and review analytic progress and all team members were involved in final agreement of the analysis.

## 4 FINDINGS

A total of 19 strategic leaders agreed to be interviewed (Australia = 6; Canada = 2; New Zealand = 1; Singapore = 1; UK = 9).

**FIGURE 1:**
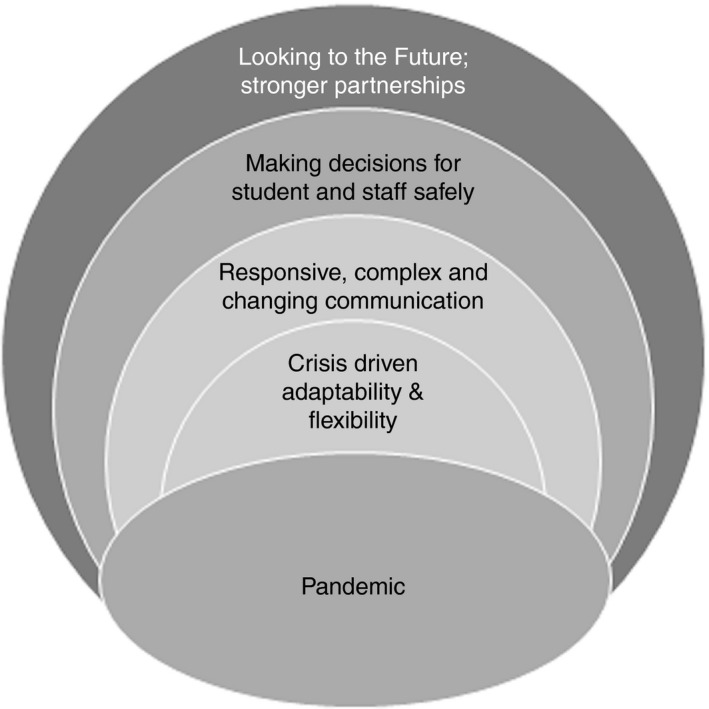
Themes

Four overarching themes arose from the analysis: (1) Crisis driven adaptability & flexibility; (2) Responsive, complex and changing communication; (3) Making decisions for student and staff safety; (4) Looking to the future; stronger partnerships. These are presented in Figure 1 below which is followed by a detailed description of each theme.

### 4.1 Crisis driven adaptability and flexibility

Although some participants reported previous experience of emergency/disaster preparation, for example, in relation to SARS and H1N1 outbreaks or state‐wide fire, none felt prepared for the complexity of COVID‐19:

To have planned for that, if I'm being entirely honest, I think would've been extraordinary, to have plans in place for something that happens every 100 years. P2

​

Not surprisingly, in the understandable absence of a plan, and given the scale of the problem, there was uncertainty and confusion about how best to respond:

So, our university was very disorganised. It couldn't make its mind up around what it wanted to do, and that was very difficult around the platform. We were going to change into block mode, and everybody was potentially going to have to reorganise their curriculum. And then there was a big revolt from the students and it hit the papers, so the university immediately backtracked. P17

​

The speed of developments also presented particular challenges with participants describing a landscape which changed significantly daily, within days and sometimes within hours:

Documents that I was working on at eight in the morning, by midday was (sic) a different document, and by six in the evening was a completely different document. And things changed on an hour by hour, sometimes minute by minute basis, and a phone call could change the direction of flow. P19

​

This required the adoption of new approaches to making decisions:

If you're dealing with something really complex which is different in different occasions, in a way you have to take the ‘must do's’ out of your standards in order to allow people to make decisions in the best interests of their students. It's almost if you don't do it that way, then you'll have a very high level, top‐down approach, which might meet the requirements of half the people but doesn't allow judgement and best solutions to be found further down. P12

​

Working in this rapidly changing situation required not only individual adaptability on the part of the leader, but also the support and flexibility of their academic teams:

The team were incredibly engaged and adapted at a pace which probably I hadn't anticipated. P3

​

The urgent need to significantly adapt the delivery of programmes brought a degree of collaboration which overrode some of the traditional competition across the sector:

I think we’ve learnt to trust each other a little bit more and share a little bit more, which has been a great outcome. And I saw evidence of that really early April when we first went into this mode, that universities were willing to share even their online skill videos with each other, and that was a great thing. That costs a lot to make those, and just to share resources I think was good, and to support each other just through those demands. P14

​

Perhaps most strikingly, crisis drove innovation, forcing providers to initiate rapid change, which under ordinary circumstances might have taken years to introduce. As a consequence of COVID‐19 rapid adaptation became the norm:

I think the higher education system as a sector has demonstrated a lot of agility. And all of the things we've been talking about for years that we needed to do, we did in a very short space of time. So, online curricula, blended learning for everybody. These are things that a university in the UK has been very much doing for a very long time, but other universities found it more difficult. But now as a result of COVID, every single university is thinking about it …So, I think COVID, like many other pandemics or many other crises, like war, the First or the Second World War became a catalyst for change. And that's not necessarily a bad thing. That's actually a good thing because it pushes people to innovation, to quick action, to delivery, to effectiveness. P7

​

This willingness to change also manifested itself in a civic response in offers of help from academic staff who volunteered to return to practice settings and in the donation of equipment and resources by universities to practice partners:

We could sort of lift our head above the parapet and say, right. What are the key things there? So, we looked at all the equipment that we had. What can we do with our equipment? So, our head technician did an inventory of what could or couldn't be moved. So, I went to the vice chancellor said this is this is equipment. We have an offer. I know that people want it. Can I have your permission to lend out this equipment and instruments? And he just turned to me and said, yes, I have to take it to the senior team. But, yes, is the answer. P5

​

### 4.2 Responsive, complex and changing communication

The first few weeks of the crisis were characterised by confusion with information arriving from multiple sources, with details subject to change at short notice.

Throughout there was confusion, miscommunication, and leaders were questioning who's making decisions, what are the priorities. In the absence of a plan, this is what you would expect. P8

​

A key priority for all participants was to collect, analyse and disseminate information as it became available. In doing this they sought to both keep students and staff informed, but also earn their trust.

It was intense but it was really important because I had to make sure that everybody felt that they knew what was happening because the staff needed to know what was happening in order to support the students and the students needed to know what was happening to make them feel secure and safe as they moved forward through the program. P11

​

While identifying the importance of a clear, consistent communication strategy as a key component of managing the crisis, for most, this was an aspiration, rather than something they felt had been fully mastered:

Communication, communication, communication. To be quicker and more effective in my role as a leader in communication with others. To develop a communication strategy rather than being ad hoc. P4

​

The rapidly evolving situation with information arriving from many sources for dissemination to multiple groups meant that communication was especially complex. It needed to be frequent and responsive, while accurately reflecting the shifting positions of government, placement providers and regulators.

And that was the other thing, we had to be very, very clear in our comms. And we had to check the comms before they went out to the students to make sure they were exact and they were right, because you had to be very, very careful in what you said. So, you had to make sure that it was right from the perspective of the government, the health service, the regulator, and from our university perspective. P13

​

The mode and medium of communication were also important, multiple different communication channels were used by the participants to deliver complex information but, also to support each other. Lack of face‐to‐face communication promoted the use of new communication platforms and channels and these helped to build a sense of community, belonging, trust and reduce anxiety.

We set up a WhatsApp group to stop the emails, because the emails were just manic. … you can say things in WhatsApp that you can't say on email. We were all on WhatsApp because you can do that, and you're going, this is mad. P13

​

### 4.3 Making decisions for student and staff safety

The rapidly unfolding nature of the pandemic mean that significant decisions often had to be taken quickly, without a full picture, and often in the absence of the usual opportunities for reflection and consultation with others. These sometimes involved balancing competing demands and risks, for example, ensuring student safety while remaining within the parameters of professional regulation and academic standards. The question of whether to continue student placements was particularly difficult, especially in the early stages when supplies of personal protective equipment were uncertain:

…we were in a difficult position because we were potentially putting our students at major risk. Because we couldn't get any guarantee that our students would be given PPE, we couldn't get any guarantee that our students would be given the same level of provision and rights as full time employees in the health service. P3

​

These very real concerns saw leaders in discussion about issues which had rarely, if ever touched them previously. One recounted details of death in service arrangements for students, while for another the stark reality of their decisions was brought home via contact with parents:

I had several parents email me and phone me up, “How could you possibly have made these decisions? How could you send my daughter into practice? If she dies, I will hold you personally responsible.” P4

​

In all cases, maintaining student safety while limiting disruption to studies was stated as the main priority:

It was always safety, was our number one … that we kept our students in areas that were safe. So, while others across the country were pulling their students out, and even my colleagues provincially here started to pull students out, we quite strategically said we will keep them in and we will keep them safe. That decision was made quickly, but we took a lot of heat on it. It took a lot of working with faculty and students to give them information of how they would be safe, how they needed to practice. P18

I think the desire to have as little disruption for the students as possible. I know that was an overriding desire for my team, very much that student focus. P1

​

One of the other major considerations of leaders was the potential impact of trauma on students who continued in placement:

So, there are areas there that we need to consider, for example student mental health services. We've had students that saw things they would have never expected to see as a student, experienced death in very difficult, extraordinary circumstances, would be right here to say. And as a result, they needed a lot more support than they would normally need in their study. So, the student mental health services need to gear up to support this group of students, because they were not like geography or history students who were sitting at home doing online lectures. There were students who were in an ICU with the dying patients, with all sorts of PPE they hadn't seen before. They were all dressed up like Darth Vader in a setting that they had never been at before, and thinking gosh, three people just died in a second, and then a hundred more are coming in, and we haven't got space for them. P7

​

The consequences of leaders’ decision making impacted on staff as well as students. As decisions were made, individuals needed to consider the effect of their actions on their teams who were also reeling from the huge disruption on lives and work patterns caused by COVID‐19. They needed to transform quickly the way they worked in terms of format and location, homeworking and the need to move provision online at pace. There was no easy way through this:

“…how do you navigate this, and how do you support a team through this? P8

​

Regardless of the who and what they were dealing with, these leaders were aware that decisions made in the here and now might have longer term consequences which could not easily be predicted and could be harmful.

You had to think of all those things together … both in terms of managing the here and now which sometimes is very easy, but also understanding immediately the consequences of our decisions and what does that mean longer term for our sector? P7

​

Participants eloquently spoke about the personal toll taken by their work, the difficult conversations and decisions they made.

…this has been the most challenging, difficult professional issue I’ve experienced. It's not going away, and it's exhausting and it's relentless. It's been really, really difficult. And, you know, we may come out the other side and we may have experienced personal growth and professional growth, but I wonder also if there will be a lot of casualties of this, because I think all the trauma, you know, in terms of workload, cognitive overload, difficult decisions. P10

​

### 4.4 Looking to the future—Stronger partnerships

Reflecting on their response to the pandemic and the context in which decisions were made, participants were able to identify areas for future development. Recognising the system is dependent on multiple stakeholders, there was an acceptance of the need for a shared vision for how to move forward beyond the immediate crisis:

I think having a really good partnership with your clinical partners and the chief nurses, accreditation bodies are absolutely critical to the management of all the moving parts. I think it's really important that all of these bodies get together as quickly as possible and develop a shared plan. P12

​

Participants also spoke about the new appetite for collaboration across the sector and indicated that its fostering and future development might also be a positive outcome:

I'm very proud about how the system came together to collaborate. It's the first time I've seen so much collaboration between regulators, government, and universities. So, that's good and I hope we can continue and maintain it for other areas as well. Those relationships are now so strong. They were a bit more formal in the past and now they've become almost more informal because of the frequency and the pace. P7

​

Many spoke about the lack of the student voice in early decision making. However, leaders were divided on the value of the student voice in these circumstances:

(student consultation?) None. We didn't have time. So, there was a bit of bitching that went on to begin with in those student town halls about, "Why weren't we consulted?" And it's like, there's nothing to consult you on. It's a public health directive that you can't be on campus, and it's the hospital's decisions that you can't be in clinical placement. So, there's nothing to consult you on. P15

​

Many leaders recognised that the consequences of their decisions and actions had an impact on the future that would be permanent:

I don't think we'll ever go back to, you know, tutorials with 20 students in face to face. That will never happen. Now we're going to have to embrace technology. We're going to use online technology more. P10

​

Although some leaders were concerned about the consequences of this, others saw opportunities to move toward a more sustainable mode of delivery:

…we're going to have another 18 months of this. You know, the knock‐on effect. So, this is about sustainability. That leads on to a totally different question about what we've learned and how can we make sure that we are sustainable. P5

​

A sense of personal regret and loss also permeated some of the interviews. Some of this related to situations which might have been dealt with differently—this was apparent in those who felt that students might have been more involved in decision making. Others were already dealing with some of hard consequences which had rapidly followed national lockdowns. By the time the interviews took place, the loss of income, particularly from international students, had already begun to hit departmental budgets with the real possibility that the colleagues who had worked so hard for students and universities would now face redundancy.

## 5 DISCUSSION

We interviewed nurse education leaders, as they sought to make sense of their unique experiences while still managing the evolving COVID‐19 pandemic. Our analysis of their interview narratives illustrates that there was a significant and important gap in policies, procedures and planning—the sector was not prepared for a crisis of this magnitude. It would be easy to criticise this evident gap in organisational planning, especially as the likelihood of a global pandemic has been discussed for several years (Morse et al., 2012). In our view, this would be unfair—developing a pandemic plan has, until now, been the responsibility of governments, not organisations, individual departments, faculties or professional and academic leaders. Considering the lack of a clear operational pandemic plan, it was evident in the analysis that the participants in our study, were adaptive and responsive, strategic decisions were made quickly, with the overarching aim of minimising risks to student health and maintaining, as best they could, the integrity of programmes and students’ progression. In order to achieve this, they were deeply and very personally involved in day‐to‐day discussions and decision making, both across the sector, with government and within their departments. They also appear to have been fully engaged with students and their parents. They managed this despite very significant increases in personal workload. To contradict Rosser et al. (2020), these leaders were indeed ‘visible’. Similarly, where Singh and Haynes (2020) have called for COVID‐19 to mark a watershed moment in terms of the need for faculty to undertake leadership training, our participants appeared to fully embrace the role of leader in this crisis. To do this they worked long hours—at home and often from make‐shift office spaces, they co‐ordinated activity, managed staff and student concerns and in doing so, sometimes found themselves crying at their desks. Making important decisions in the absence of full data sets and at pace, they drew on each other for support, created new platforms of communication and developed stronger and more collaborative partnerships with clinical providers and each other.

We identified and it has been argued that students and frontline staff may require ongoing support following their pandemic experiences (Nelson & Lee‐Winn, 2020; Savistsky et al., 2020). We suggest that it would also be prudent to consider how those in leadership positions might best be supported. Our analysis of the participants’ transcripts revealed the complex, challenging but essential role that nurse education leaders play in promoting student safety, developing the nursing profession and ensuring a workforce pipeline that is fit for practice. Our findings highlight the vital role nursing education plays in providing an ongoing competent workforce that is imperative to health care delivery.

To manage the rapidly changing situation, nurse education leaders in our study collaborated with competitors with whom they shared ideas, intelligence and resources, and also looked to for support. Given the reality in some areas, and strong likelihood in others, that educational budgets will be severely impacted by the economic shock of COVID‐19, one way in which this might be mitigated would be to foster that collaborative spirit. Alongside this is a real opportunity to overhaul the way in which nurse education programmes are provided. The pandemic has forced varying degrees of increased online learning upon the sector (Konrad et al., 2020). There has not been time to evaluate these, but it seems likely that they are here to stay (Morin, 2020). To make the most of this opportunity, universities will need to review their information technology infrastructure and consider the degree to which it is ready for this future.

Nurse education leaders may not work at the coalface of health care—although some still do, but their role and contribution to the pandemic has been significant. The analysis presented in this paper illustrates the vital role they played in driving health care policy but more importantly protecting student nurses and delivering a competent educated workforce for the future.

### 5.1 Limitations

As with all qualitative work, we accept that our analysis cannot represent the views of all those who provided nurse education leadership. Those that did not reply to the invitation be involved may have had a very different experience than we have presented. We are also aware that while one of the strengths of our work is that it captured leaders’ perspectives in the moment of crisis, time and distance might have allowed them space to process, make sense of and detail their experience differently. Finally, we must acknowledge that researchers as insiders to the domain under investigation and as participants may have impacted the analysis and findings.

## 6 CONCLUSIONS

Internationally, while nursing education leaders faced different problems, they shared a common goal amidst the crisis to remain student‐centred. This meant balancing the desire to keep students safe against enabling them to complete their programmes of study in a timely manner and taking account of regulatory requirements. This appeared to stem from both a desire to support students and provide for the ongoing needs of the healthcare communities for which graduate nurses are critical. Recognition that the pandemic is far from over places emphasis on the adaptability and transformation of nursing programs to continue to be able to meet demand for nursing workforce requirements.

The nurse leaders in our study, demonstrated they were able to face major challenges, respond to large scale logistical problems and make decisions under significant and ongoing pressure. Looking to the future, nursing leaders are ideally positioned to review the ‘lessons learned’ from the multitude of student and course related issues that emerged during the pandemic and to use these to inform the development of future nursing programmes, that perhaps better reflect learning needs of students and the practice needs of modern health services.

## AUTHOR CONTRIBUTION

RI, DM, AC, AJ, LH, LK,NH‐M: made substantial contributions to conception and design, or acquisition of data, or analysis and interpretation of data; RI, DM, AC, AJ, LH, LK,NH‐M: involved in drafting the manuscript or revising it critically for important intellectual content; RI, DM, AC, AM, LH, LK,NH‐M: given final approval of the version to be published. Each author should have participated sufficiently in the work to take public responsibility for appropriate portions of the content; RI, DM, AC, AJ, LH, LK,NH‐M: agreed to be accountable for all aspects of the work in ensuring that questions related to the accuracy or integrity of any part of the work are appropriately investigated and resolved.

### PEER REVIEW

The peer review history for this article is available at https://publons.com/publon/10.1111/jan.14892.

## Data Availability

Research data are not shared.
